# BTBD7 Downregulates E-Cadherin and Promotes Epithelial-Mesenchymal Transition in Lung Cancer

**DOI:** 10.1155/2019/5937635

**Published:** 2019-11-27

**Authors:** Jie Shu, Lin Wang, Fei Han, Yubin Chen, Shunjun Wang, Fanyan Luo

**Affiliations:** Department of Cardiothoracic Surgery, Xiangya Hospital, Central South University, Changsha 410008, Hunan, China

## Abstract

Metastasis is the leading cause of lung cancer-associated death. Downregulated expression of E-cadherin followed by epithelial-mesenchymal transition (EMT) is critical for metastasis initiation in lung cancer. BTBD7 plays essential roles in lung cancer metastasis, but the mechanisms remain unknown. This study aimed to investigate the relationship between BTBD7 and E-cadherin in lung cancer and explore the role of BTBD7 in EMT. Fresh lung cancer and paracancer tissue specimens were collected from 30 patients, and the expression of BTBD7, E-cadherin, N-cadherin, fibronectin, and vimentin was analyzed by qRT-PCR, western blotting, and immunohistochemistry. A549 and HBE cells were cultured and treated with TGF-*β*1 for 72 h to induce EMT. Western blotting and qRT-PCR were performed to evaluate the expression of BTBD7, E-cadherin, N-cadherin, fibronectin, and vimentin. Then, A549 cells were treated separately with the BTBD7-ENTER plasmid, BTBD7-siRNA, and paclitaxel. After TGF-*β*1-induced EMT, the abovementioned markers were analyzed by western blotting and qRT-PCR. Wound healing assays were applied to assess the migration ability of cells in different groups. For animal experiments, A549 cells transfected with the BTBD7-ENTER plasmid were transplanted into BALB/c nude mice. After 4 weeks, all nude mice were sacrificed, and tumor tissues were harvested for qRT-PCR, western blot, and immunohistochemical analyses of the abovementioned markers. All experimental results showed that the levels of BTBD7, N-cadherin, fibronectin, and vimentin were increased in lung cancer tissues and cells, while the E-cadherin level was decreased. Transfection experiments showed that BTBD7 inhibited E-cadherin expression and enhanced EMT. Moreover, the migration capacity of lung cancer cells was increased by the high level of BTBD7. We concluded that BTBD7 is highly expressed during lung cancer development and metastasis and can inhibit the expression of E-cadherin and promote EMT in lung cancer. BTBD7 may thus be a therapeutic target for lung cancer.

## 1. Introduction

Lung cancer is a leading cause of cancer-related death worldwide, accounting for approximately 28% of cancer-related deaths [1–3]. Currently, although great progress has been made in the prevention and treatment of cancer, the mechanism underlying cancer is still unclear, the rate of cancer mortality remains high, and the 5-year survival rate is quite low—only 17% [3]. Recurrence, metastasis, and drug resistance are common causes of lung cancer treatment failure. Therefore, studying the mechanism of lung cancer development and developing new targeted drugs to block the development of lung cancer are of great importance in improving the cure rate and survival rate of lung cancer patients.

Epithelial-mesenchymal transition (EMT) refers to the phenotypic transformation of epithelial cells to mesenchymal cells under specific physiological and pathological conditions, accompanied by changes in cell morphology and cell function [4]. EMT has a common cytological mechanism: proteins associated with epithelial properties, such as E-cadherin and *β*-catenin, are downregulated, and proteins associated with mesenchymal properties, such as vimentin, fibronectin (FN), and N-cadherin, are upregulated; *β*-catenin enters the nucleus; and cells then undergo cytoskeletal changes, thus gaining a strong capacity for migration [5–7]. EMT plays an important role in normal physiological processes, such as embryonic development and tissue damage repair. During tumor development, epithelial-derived malignant cells lose their tight junction with neighboring cells through EMT, especially the reduction in E-cadherin, to escape the primary tumor, and they acquire the migratory characteristics of mesenchymal cells and migrate to the tumor matrix. Thus, EMT is a key initial step in the process of tumor invasion and metastasis [8]. Numerous clinical studies have confirmed that the expression of EMT markers is closely related to metastasis and prognosis in cancer patients [9, 10]. Therefore, elucidating the molecular mechanism of EMT regulation in malignant tumor cells and exploring targeted therapies based on key EMT molecules are an important topic of cancer research.

BTB/POZ domain-containing protein 7 (BTBD7) is a highly conserved protein that mainly functions to mediate protein-protein interactions. BTB/POZ domain proteins have diverse biological functions in eukaryotic cells in processes such as tissue and organ development, apoptosis, protein degradation, cell movement, and tumor formation [11, 12]. BTBD7 was first reported in 2001 as a liver cancer-related gene that promotes cell proliferation and tumor formation [13, 14]. Transfection of BTBD7 into Madin–Darby canine kidney (MDCK) cells revealed that expression of E-cadherin was decreased in MDCK cells, cell junctions became loose, and migration capacity increased [15]. Fan et al. [16] reported that BTBD7 is associated with downregulation of E-cadherin and poor prognosis in non-small cell lung cancer. After transfection of lung cancer cells with BTBD7-siRNA, the expression of E-cadherin was upregulated, and the cell migration ability was decreased [16]. These studies suggest that BTBD7 is likely to be a novel key molecule in the regulation of E-cadherin. Therefore, we hypothesized that BTBD7 can downregulate E-cadherin and promote EMT in lung cancer cells and that it is linked to the development of lung cancer. The molecular mechanism of EMT regulation in lung cancer will be studied further; this study will help to elucidate the mechanism of lung cancer development and lay a theoretical foundation for the development of novel targeted drugs.

## 2. Materials and Methods

### 2.1. Ethical Considerations

All protocols described in this study were approved by the Xiangya Hospital Ethics Committee and carried out in accordance with the Declaration of Helsinki. Informed consent was obtained from all participants and/or their legal guardian/s.

### 2.2. Changes in BTBD7 during TGF-*β*1-Induced EMT of Lung Cancer Cells

A549 and HBE cells were cultured in RPMI-1640 medium (Thermo Scientific, Waltham, MA, USA) supplemented with 10% FBS. After the cells were more than 85% confluent, they were cultured in serum-free medium for approximately 6 h, and TGF-*β*1 (Merck KGaA, Darmstadt Germany) at a final concentration of 20 ng/ml was then added to treat the cells. After 72 h of culture, total RNA and protein were collected. The levels of BTBD7, E-cadherin, and EMT markers were analyzed by quantitative reverse transcription-PCR (qRT-PCR) and western blotting.

### 2.3. Effects of BTBD7 Silencing and Overexpression on E-Cadherin and EMT

To further investigate the effect of BTBD7 on E-cadherin and EMT, BTBD7 overexpression plasmids and the corresponding control plasmids and interference plasmids and the corresponding control plasmids were transfected into A549 cells using a Lipofectamine 2000 (Invitrogen, 11668-019) transfection kit. We also added paclitaxel (Shanghai Yuanye Biotechnology Co., Ltd, 33069-62-4) at a final concentration of 10 nM to transfected cells to evaluate its effect on BTBD7. The groups were divided as follows: A, the BTBD7-ENTER group (overexpression plasmid group); B, the p-ENTER group (control plasmid group); C, the BTBD7-siRNA group; D, the scrambled siRNA group; E, the BTBD7-ENTER + paclitaxel group; F, the BTBD7-siRNA + paclitaxel group; and NC, the HBE group. After 24 h of culture, TGF-*β*1 was used to induce EMT in every group. In groups E and F, after EMT induction, paclitaxel was added to treat cells for 48 h. Then, all samples were collected, and BTBD7, E-cadherin, and EMT markers were analyzed by western blotting and qRT-PCR. BTBD7-ENTER (NM-018167) and p-ENTER (PD88001), as well as BTBD7-siRNA (HM3262-B09-U6) and scrambled siRNA (plent-U6–puro), were obtained from Vigene Biosciences (USA).

### 2.4. In Vivo Study

An animal model of lung cancer metastasis was established using BALB/c nude mice to study the role of BTBD7 in promoting EMT and metastasis in lung cancer. Animals were obtained from Hunan SJA Laboratory Animal Co., Ltd. (SCXK2016-0002). A549 cells were transfected with BTBD7-ENTER plasmid and were then inoculated subcutaneously into 3 nude mice. Another 3 nude mice were transfected with BTBD7-p-ENTER plasmid as the control group. All animals were sacrificed 4 weeks later. The tumor tissues were harvested, and qRT-PCR and western blotting were applied to analyze the expression of BTBD7, E-cadherin, and EMT markers. Immunohistochemistry was utilized to detect BTBD7 expression.

### 2.5. Western Blot Analysis

We used RIPA lysis buffer (Beyotime, China) to lyse cells and tissues. The concentrations of total protein were detected with a BCA Protein Assay Kit (TaKaRa). SDS-PAGE gels (10%) were used to separate proteins, and after electrophoresis, proteins were transferred to PVDF membranes. Then, membranes were blocked with 5% BSA. After blocking, membranes were incubated with rabbit polyclonal anti-human BTBD7 (ab154685; Abcam, 1 : 1000 dilution), rabbit polyclonal anti-human E-cadherin (ab133597; Abcam, 1 : 4000 dilution), rabbit polyclonal anti-human N-cadherin (ab76057; Abcam, 1 : 1000 dilution), rabbit polyclonal anti-human FN (ab76057; Abcam, 1 : 1000 dilution), and rabbit polyclonal anti-human vimentin (ab137321; Abcam, 1 : 2000 dilution) antibodies and with anti-*β*-actin (ab8227, Abcam, 1 : 3000 dilution) as the loading control. Each membrane was subsequently incubated with the corresponding secondary antibodies. Finally, we used an enhanced chemiluminescence (ECL) kit to visualize protein bands in a ChemiDoc XRS Plus luminescence image analyzer (Bio-Rad). The results are shown as relative grayscale values.

### 2.6. qRT-PCR

We used Trizol (TaKaRa) to extract total RNA from cells and tissues, and reverse transcription was performed by using a Bio-Rad iScript™ cDNA Synthesis Kit. A template equivalent of 500 ng of total RNA was subjected to 45 cycles of quantitative PCR (qPCR) with All-In-One qPCR Mix (GeneCopoeia, America) on a CFX96TM Real-Time PCR Detection System (Bio-Rad). We adopted *β*-actin as an internal reference. The results are shown as the relative quantitation (RQ) values. The following primer sequences were used for expression analysis: BTBD7, forward primer: 5′AGACGCCTTCGACCATCAC3′, reverse primer: 5′ CTCCCCTAGCTGGGTTGTAGA3′; E-cadherin, forward primer: 5′ CGAGAGCTACACGTTCACGG3′, reverse primer: 5′ GGGTGTCGAGGGAAAAATAGG3′; N-cadherin, forward primer: 5′ TTTGATGGAGGTCTCCTAACACC3′, reverse primer: 5′ ACGTTTAACACGTTGGAAATGTG3′; FN, forward primer: 5′ TCTGTGCCTCCTATCTATGTGC3′, reverse primer: 5′ GAGGGACCACGACAACTCTTC3′; and vimentin, forward primer: 5′ GACGCCATCAACACCGAGTT3′, reverse primer: 5′ CTTTGTCGTTGGTTAGCTGGT3′.

### 2.7. Immunohistochemical Analysis

Specimens were fixed in standard 10% neutral formaldehyde solution, embedded in paraffin, sliced to a thickness of 4 *μ*m, dewaxed and rehydrated, and subjected to microwave antigen retrieval in sodium citrate buffer (pH 6.0). Endogenous peroxidase activity was blocked by hydrogen peroxide treatment. Nonimmune animal serum was used to block nonspecific reactions. Rabbit anti-human BTBD7 monoclonal antibody was added dropwise, incubated for 1 h at room temperature, and then incubated overnight at 4°C. After slides were rewarmed at 37°C for 1 h, biotin-labeled secondary antibody was added. Then, DAB was added for color development, and slides were washed with tap water and dehydrated in 70%–100% reagent alcohol baths followed by xylene baths before coverslips were applied. Finally, slides were evaluated using a microscope.

### 2.8. Wound Healing Assay

Cells were seeded in 6-well plates at a concentration of 1 × 10^5^ cells/mL. At 90% confluence, the cells were scratched with a micropipette tip (200 *μ*L). A single scratch of approximately 1 mm × 10 mm (width × length) was made in each well. The vicinity of the scratch was gently washed with serum-free medium, and the medium was aspirated; this process was repeated 3 times. The scraped cells were then rinsed as thoroughly as possible. After 6 and 24 h of culture, the 6-well culture plate was placed under an inverted microscope, and the migration of cells around the scratch to the damaged area was observed. The closure of the scratched area was imaged and measured for statistical analysis.

### 2.9. Statistical Analysis

Differences in data between groups were compared by SPSS 19.0 with one-way analysis of variance (ANOVA) or unpaired Student's *t*-test. For ANOVA, if the variance was significant, the Bonferroni or least significant difference (LSD) test was applied for post hoc analysis. All data are presented as the means ± standard errors of the mean (SEMs), and *p* < 0.05 was considered statistically significant.

## 3. Results

### 3.1. Expression of BTBD7 and EMT Markers Is Increased in Cancer Tissues, while E-Cadherin Expression Is Decreased

**Figure 1:**
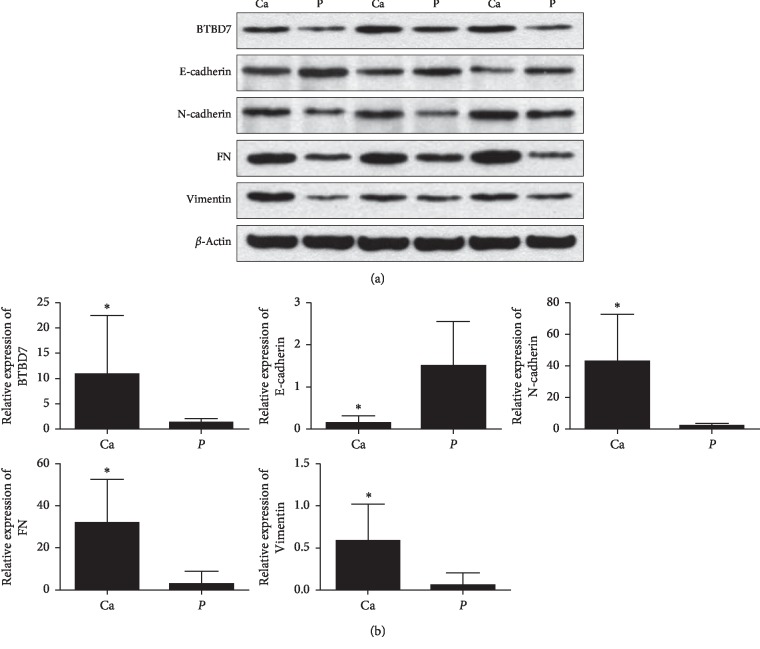
Relative protein and mRNA levels of BTBD7, E-cadherin, N-cadherin, FN, and vimentin. Ca: lung cancer tissue (*n* = 30). P: Paracancer tissue (*n* = 30). The bars indicate the mean values±SDs. ^*∗*^*p* < 0.05.

Lung cancer and paracancer tissues were obtained from patients for western blotting. The relative protein levels of BTBD7 (0.45 ± 0.16), FN (0.81 ± 0.24), and vimentin (0.42 ± 0.21) in lung cancer tissues were significantly higher (*p* < 0.05) than those in paracancer tissues (0.20 ± 0.09, 0.26 ± 0.16, and 0.15 ± 0.13, respectively). In addition, the expression of E-cadherin (0.20 ± 0.11) in lung cancer tissues was significantly lower than that in paracancer tissues (0.45 ± 0.15) (*p* < 0.05). However, the expression of N-cadherin was not significantly different between lung cancer (0.40 ± 0.21) and paracancer tissues (0.34 ± 0.24) (*p* < 0.05) (Figure 1(a)).

The qRT-PCR results showed that the expression of BTBD7 (10.76 ± 11.71), N-cadherin (42.48 ± 30.17), FN (31.93 ± 20.69), and vimentin (0.58 ± 0.44) in lung cancer tissues was significantly higher than that in paracancer tissues (1.16 ± 0.63, 2.84 ± 6.05, 2.84 ± 6.05, and 0.06 ± 0.14, respectively) (*p* < 0.05). Conversely, the level of E-cadherin in lung cancer tissues (0.13 ± 0.17) was significantly lower than that in paracancer tissues (1.50 ± 1.15) (*p* < 0.05) (Figure 1(b)).

**Figure 2:**
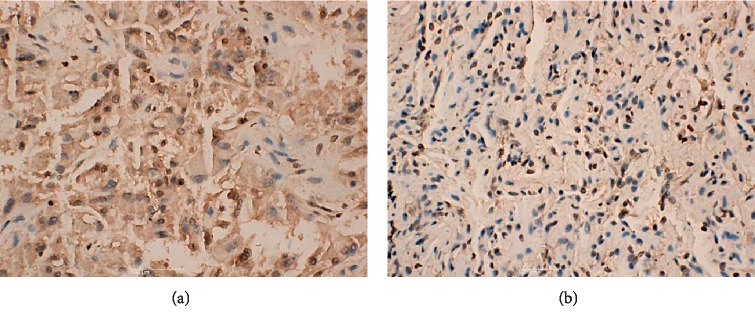
Immunohistochemical analysis of BTBD7. The brown areas indicate positive staining. (a) Lung cancer tissue (*n* = 30). (b) Paracancer tissue (*n* = 30).

Immunohistochemical analysis showed significantly larger regions with positive BTBD7 staining in lung cancer tissues than in paracancer tissues (Figure 2).

### 3.2. Effect of BTBD7 on TGF-*β*1-Induced EMT in Lung Cancer Cells

**Figure 3:**
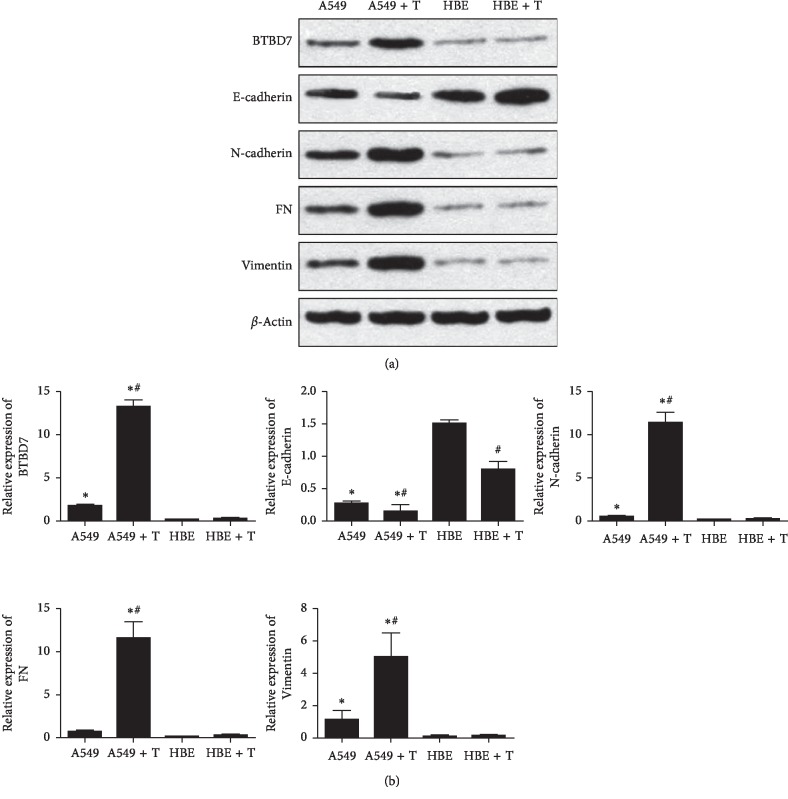
Relative protein and mRNA levels of BTBD7, E-cadherin, and EMT markers in different groups. The bars indicate the mean values±SDs. (^*∗*^A549 groups compared with HBE groups, *p* < 0.05. #TGF-*β*1-treated groups compared with untreated groups, *p* < 0.05.)

After measuring the expression of BTBD7, E-cadherin, and other EMT markers in clinical specimens, we further studied the role of BTBD7 in EMT in vitro. After TGF-*β*1 induction, western blot analysis showed that the expression of BTBD7 (1.05 ± 0.34), N-cadherin (1.34 ± 0.25), FN (1.10 ± 0.07), and vimentin (13.22 ± 0.86) in A549 cells was significantly higher than that in HBE cells (0.16 ± 0.13, 0.05 ± 0.002, 0.05 ± 0.04, and 0.27 ± 0.03, respectively) (*p* < 0.05), while the expression of E-cadherin (0.25 ± 0.10) was significantly lower in A549 cells than in HBE cells (0.99 ± 0.09) (*p* < 0.05). In addition, the expression of BTBD7, N-cadherin, FN, and vimentin in TGF-*β*1-treated A549 cells was significantly higher than in untreated A549 cells (0.42 ± 0.25, 0.50 ± 0.03, 0.28 ± 0.01, and 1.79 ± 0.13, respectively) (*p* < 0.05). The level of E-cadherin showed the opposite trend, and the difference between treated A549 cells (0.25 ± 0.10) and untreated A549 cells (0.50 ± 0.05) was statistically significant (*p* < 0.05). However, the difference in the expression level of each protein between TGF-*β*1-treated HBE cells and control HBE cells was not statistically significant (*p* > 0.05). The western blot results showed that BTBD7 showed a tendency toward high expression during the process of EMT in lung cancer cells (Figure 3(a)).

The mRNA expression levels of these markers were measured by qRT-PCR and showed a trend similar to that shown by western blot analysis (Figure 3(b)).

### 3.3. BTBD7 Inhibits the Expression of E-Cadherin and Increases the Expression of Other EMT Markers

**Figure 4:**
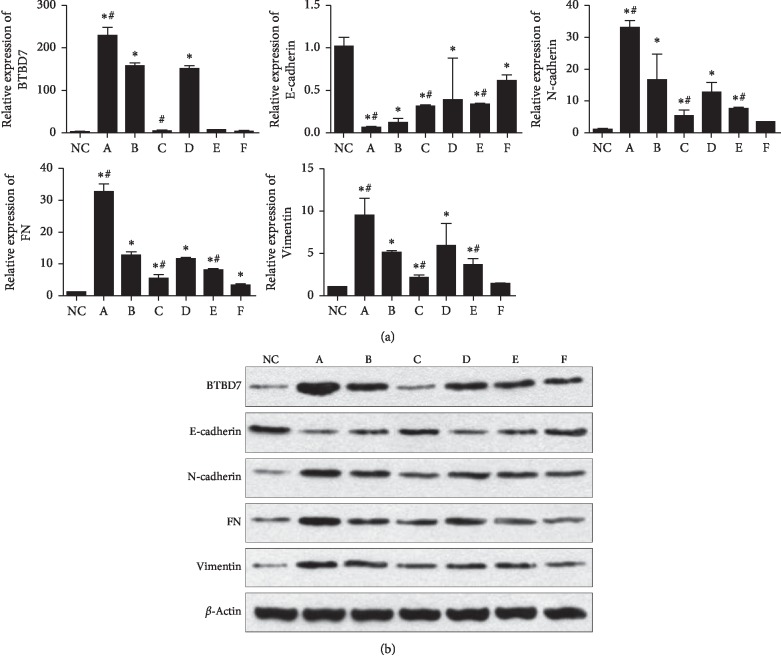
Relative mRNA and protein levels of BTBD7, E-cadherin, and EMT markers in A549 and HBE cells: (a) BTBD7-ENTER group; (b) p-ENTER group; (c) BTBD7-siRNA group; (d) scrambled siRNA group; (e) BTBD7-ENTER + paclitaxel group; (F) BTBD7-siRNA + paclitaxel group. NC: HBE group. The bars indicate the mean values±SDs. (^*∗*^significant difference compared with the NC group, *p* < 0.05. #significant difference compared with the internal control group, *p* < 0.05.)

To further investigate the effect of BTBD7 on E-cadherin and EMT markers, we transfected A549 cells with the BTBD7 overexpression plasmid and BTBD7-siRNA and used paclitaxel to treat transfected A549 cells to evaluate whether the drug could influence the expression of BTBD7. qRT-PCR showed that the expression of BTBD7 in the BTBD7-ENTER group (228.09 ± 20.56) was significantly higher than that in the p-ENTER group (157.26 ± 6.81) (*p* < 0.05). The expression levels of EMT markers were increased significantly in A549 cells overexpressing BTBD7, while the expression of E-cadherin was significantly decreased in the BTBD7-ENTER group (0.06 ± 0.01) compared with the p-ENTER group (0.12 ± 0.05) (*p* < 0.05). The expression pattern of these genes in the BTBD7-siRNA group, and the scrambled siRNA group was opposite than that in the former two groups. In the paclitaxel groups, paclitaxel inhibited BTBD7 in both BTBD7-overexpressing (6.39 ± 0.16) and BTBD7-silenced (2.34 ± 1.03) A549 cells (*p* < 0.05), but the effect was more obvious in BTBD7-silenced cells (Figure 4(a)).

The trend in the western blot results was basically the same as that in the qRT-PCR results, indicating that increased expression of BTBD7 can downregulate the expression of E-cadherin and simultaneously regulate the expression of EMT-related markers, thus promoting EMT (Figure 4(b)).

### 3.4. Wound Healing Assay

**Figure 5:**
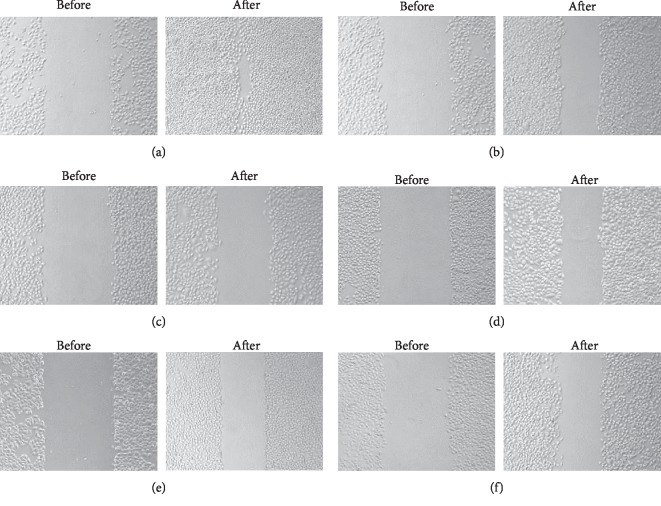
Cell wound healing assay: (a) BTBD7-ENTER group; (b) p-ENTER group; (c) BTBD7-siRNA group; (d) scrambled siRNA group; (e) BTBD7-ENTER + paclitaxel group; (f) BTBD7-siRNA + paclitaxel group (NC: HBE group).

All groups of cells exhibited wound closure. However, the BTBD7-ENTER group had a significantly higher healing rate than the p-ENTER group (*p* < 0.05). The healing rate in the BTBD7-siRNA group was significantly lower than that in the scrambled siRNA group. The healing rate in the BTBD7-ENTER + paclitaxel group was nonsignificantly lower than that in the BTBD7-siRNA + paclitaxel group (*p* > 0.05). The wound healing assay demonstrated that the migration of A549 lung cancer cells was regulated by BTBD7 and that targeted interference with BTBD7 could affect the metastasis of lung cancer cells (Figure 5).

### 3.5. BTBD7 Inhibits the Level of E-Cadherin and Increases the Levels of EMT Markers In Vivo

**Figure 6:**
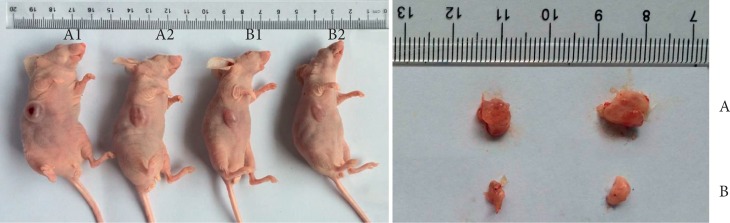
Tumor formation in nude mice: (a) BTBD7-ENTER group (*n* = 3); (b) p-ENTER group (*n* = 3).

A549 cells were transfected with the BTBD7 BTBD7-ENTER and p-ENTER plasmids. Then, these cells were transfected into subcutaneous tissue of nude mice. The tumorigenesis results are shown in Figure 6. All tumors in the BTBD7-ENTER group were larger than those in the p-ENTER group, indicating that BTBD7 enhanced the development of lung cancer.

**Figure 7:**
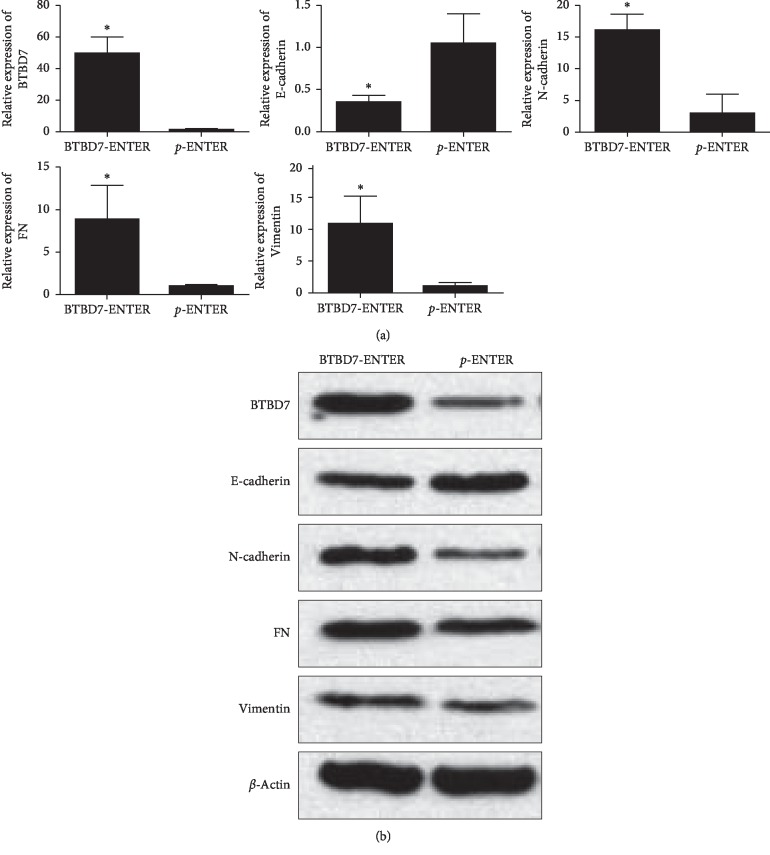
Relative mRNA and protein levels of BTBD7, E-cadherin, N-cadherin, FN, and vimentin in tumors of the BTBD7-ENTER group (*n* = 3) and p-ENTER group (*n* = 3) of nude mice (^*∗*^differences between the BTBD7-ENTER group and the p-ENTER group were statistically significant, *p* < 0.05).

The results of qRT-PCR on the tumor tissues showed that the expression of the BTBD7 (49.37 ± 10.84), N-cadherin (16.1 ± 2.61), FN (8.80 ± 4.03), and vimentin (10.82 ± 4.41) genes in the BTBD7-ENTER group was significantly higher than that in the p-ENTER group (1.05 ± 0.37, 2.89 ± 3.02, 1.00 ± 0.09, and 1.08 ± 0.52, respectively) (*p* < 0.05). The expression of E-cadherin in the BTBD7 group (0.34 ± 0.08) was significantly lower than that in the p-ENTER group (1.05 ± 0.36) (*p* < 0.05) (Figure 7(a)).

The western blot results showed the same trends in BTBD7, E-cadherin, N-cadherin, FN, and vimentin expression in the BTBD7-ENTER and p-ENTER groups as shown by qRT-PCR. Overexpression of BTBD7 in lung cancer cells demonstrated a significant downregulation of E-cadherin expression and upregulated expression of EMT markers in vivo (Figure 7(b)).

**Figure 8:**
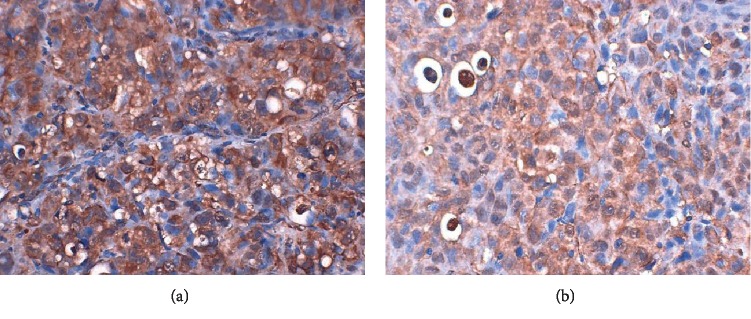
Immunohistochemical analysis of BTBD7 (*n* = 3). The brown areas represent positive staining. (a) BTBD7-ENTER group. (b) p-ENTER group.

Immunohistochemical analysis showed significantly larger regions with positive BTBD7 staining in the BTBD7-ENTER group than in the p-ENTER group (Figure 8).

## 4. Discussion

Lung cancer, which is a serious threat to human health, is the most common malignant tumor and has the highest incidence and mortality rates among cancers. Because lung cancer is asymptomatic in early stages and lacks effective screening methods, only 19% of lung cancer patients are diagnosed with localized lesions [17], and most patients have lost the opportunity for surgery by the time of diagnosis. Although multimodal treatment of surgery combined with radiotherapy and chemotherapy has achieved certain effects, the 5-year survival rate of patients is still very low [18]. EMT plays a very important role in the progression of lung cancer and is closely related to tumor metastasis and drug resistance [19, 20]. In lung cancer A549 cells, TGF-*β* can induce EMT and promote tumor migration and invasion [21]. Thus, TGF-*β*-induced EMT in lung cancer cells is an important biological mechanism of lung cancer development [22]. During EMT, epithelial cells lose their polarity and transform into irregularly shaped mesenchymal cells with changes in related molecular markers. In our study, A549 cells that underwent TGF-*β*1-induced EMT showed increased expression of BTBD7 and EMT markers but decreased expression of E-cadherin. E-cadherin is a calcium-dependent transmembrane glycoprotein that mediates tight junctions between epithelial cells and maintains the polarity of epithelial cells. When E-cadherin is downregulated, epithelial cells lose their tight junctions, and EMT then occurs. Therefore, the mechanism of E-cadherin downregulation is a key topic of EMT research. In the present study, we demonstrated that the level of E-cadherin is negatively related to BTBD7 expression. However, the specific mechanism of E-cadherin downregulation is still unclear, but it involves multiple aspects, such as gene mutation, transcription, translation, and modification and protein stability. Various endogenous and exogenous mediators of EMT in tumor cells, including p53 mutation, hypoxia-inducible factor (HIF), TGF-*β*, transcription factors (including Snail1, Snail2, Zeb1, Zeb2, and Twist), and some microRNAs, especially the miR-200 family, can downregulate the expression of E-cadherin via their corresponding pathways [23].

BTBD7 contains 1130 amino acid residues and two BTB/POZ domains, and it is localized in both the nucleus and cytoplasm. The BTB/POZ domain consists of approximately 100 amino acid residues, but its amino acid sequence is highly variable, forming a spatial conformation with a unique three-dimensional fold and a large contact plane. BTBD7 is closely related to the process of EMT. By downregulating E-cadherin and promoting epithelial cell movement, BTBD7 plays a key regulatory role in the development of the branching morphology of salivary glands and lungs. Onodera et al. [15] found that FN can induce the expression of BTBD7 in embryonic salivary gland epithelial cells. Studies have shown that FN binding to tumor cell membrane integrins can activate downstream signaling pathways such as the integrin-linked kinase (ILK) and NF-κΒ pathways, thereby regulating the expression of EMT-related genes in lung cancer [24, 25]. However, since BTBD7 does not contain a DNA-binding domain, it does not directly regulate the transcription of E-cadherin. Moreover, in lung cancer, BTBD7 is localized mainly in the cytoplasm; thus, it likely downregulates E-cadherin through regulation of protein levels. However, except for the BTB family's common binding protein E3 ubiquitin ligase complex [26], no other information about binding to the BTBD7 protein has been reported, and this topic needs further research.

Activation of transforming growth factor-*β* (TGF-*β*) often affects the tumor microenvironment. Disruption of cadherin/catenin complexes induced by these stimulations yields aberrant extracellular matrix (ECM) production and characteristics of epithelial-mesenchymal transition (EMT) [27]. Cancer cells undergo significant lipid metabolic reprogramming to ensure sufficient energy supply for survival and progression. In a present study, Wang et al. [28] demonstrated that C/EBP*δ*, a critical lipid metabolic regulator, is a TGF-*β*1 downstream gene and promotes lung adenocarcinoma metastasis. Importantly, C/EBP*δ* caused significant oscillations in both lipid metabolic and epithelial to mesenchymal transition (EMT) gene networks. Mechanistically, they demonstrated that C/EBP*δ* recruited oncogene NCOA3 to transcriptionally activate Slug, a canonical EMT transcription factor, which in turn induced oxLDL receptor-1 (Lox1) expression and enhanced oxLDL uptake to promote cancer metastasis, which could be blocked with LOX1 neutralizing antibody. Additionally, Li et al. [29] demonstrated that heterogeneous ribonucleoproteins (hnRNPs) are involved in the metastasis-related network. HnRNP K can promote the EMT process of lung *cancer* cells induced by TGF-*β*1 through interaction with MAP 1B-LC1. The interaction of MAP 1B/LC1 with hnRNP K may improve our understanding on the mechanism of TGF-*β*1-induced EMT in lung cancer.

In summary, we found that BTBD7 is highly expressed in lung cancer tissues and A549 cells and induces EMT mainly by downregulating E-cadherin and upregulating N-cadherin, vimentin, and FN. In this study, we first used an animal model to demonstrate this process. All clinical, cell-based, and animal experiments demonstrated the same results; thus, BTBD7 may be an important therapeutic target in lung cancer.

## 5. Conclusions

BTBD7 is expressed at a high level during the development and metastasis of lung cancer and inhibits the expression of E-cadherin and promotes EMT in lung cancer. Moreover, BTBD7 enhances the migration ability of A549 cells. BTBD7 may thus be a therapeutic target for lung cancer.

## Data Availability

The datasets generated and analyzed during this study are available from the corresponding author upon reasonable request.
